# Long exposure of extracellular vesicles of cerebrospinal fluid from patients with Alzheimer's disease induce the pathology in C57BL/6 mice

**DOI:** 10.1002/alz70855_100858

**Published:** 2025-12-23

**Authors:** Maria Capdevila, Laura Guzmán, Marina Carrasco, Raquel Puerta, Laura Montrreal, Montserrat Alegret, Marta Marquié, Mercè Boada, Antonio Camins, Agustin Ruiz, Miren Ettcheto Arriola, Amanda Cano

**Affiliations:** ^1^ Ace Alzheimer Center Barcelona, Barcelona, Spain; ^2^ University of Barcelona, Barcelona, Spain; ^3^ Ace Alzheimer Center Barcelona‐Universitat Internacional de Catalunya, Barcelona, Spain; ^4^ Ace Alzheimer Center Barcelona, Barcelona, Barcelona, Spain; ^5^ University of Texas Health Science Center, San Antonio, TX, USA

## Abstract

**Background:**

Extracellular vesicles (EVs) have been described to be involved in the pathogenesis of Alzheimer's disease (AD), but the mechanism remain uncertain. This study aims to examine the AD spreading role of EVs through the exposure of isolated EVs from the cerebrospinal fluid (CSF) of patients with AD to wild type mice.

**Methods:**

CSF EVs from subjects with AD dementia were administered to C57BL/6 mice for 3 months. The Morris water maze (MWM) and Novel object recognition (NOR) tests were selected to evaluate the spatial memory deficits. Neuroinflammation and synaptic integrity were evaluated with GFAP/Iba1 and synaptophysin immunohistochemistry assays. Aβ plaques were analyzed using Thioflavin‐S (ThS) staining. Proteomic profiles of human CSF EVs and mice plasma were analyzed by using CNS NULISAseq™ (Alamar BioSciences).

**Results:**

Animals exposed to AD CSF EVs showed significant memory deficits in both MWM (*p* = 0.0207) and NOR (*p* = 0.0394) tests compared to their control littermates. Immunohistochemical analysis showed significant differences in the neuroinflammation and synaptic pattern in AD EVs‐exposed animals. ThS staining detected no Aβ plaques in either group. However, proteomic analysis revealed significant differences in the soluble fractions in both human CSF EVs and mice plasma between AD dementia and control groups.

**Conclusion:**

Preliminary results suggest that EVs may play a key role in the dissemination of AD pathology. Long‐exposure of AD CSF EVs to healthy mice appears to affect their cognitive function, learning and memory ability and promotes neuroinflammation and synaptic impairment. Further experiments are still needed for a better understanding of these effects.

